# Pomegranate Peel Extract Prevents Bone Loss in a Preclinical Model of Osteoporosis and Stimulates Osteoblastic Differentiation *in Vitro*

**DOI:** 10.3390/nu7115465

**Published:** 2015-11-11

**Authors:** Mélanie Spilmont, Laurent Léotoing, Marie-Jeanne Davicco, Patrice Lebecque, Elisabeth Miot-Noirault, Paul Pilet, Laurent Rios, Yohann Wittrant, Véronique Coxam

**Affiliations:** 1 Unité de Nutrition Humaine, CRNH Auvergne, UMR 1019, INRA, F-63000 Clermont-Ferrand, France; mel.spilmont@gmail.com (M.S.); laurent.leotoing@clermont.inra.fr (L.L.); marie-jeanne.davicco@clermont.inra.fr (M.-J.D.); Patrice.Lebecque@clermont.inra.fr (P.L.); yohann.wittrant@clermont.inra.fr (Y.W.); 2 Unité de Nutrition Humaine, Université d'Auvergne, Clermont Université, BP 10448, F-63000 Clermont-Ferrand, France; 3 GREENTECH SA Biopôle Clermont-Limagne, F-63360 Saint-Beauzire, France; laurent.rios@vetagro-sup.fr; 4 Imagerie Moléculaire et Thérapie Vectorisée, Université d'Auvergne, Clermont Université, UMR 990, INSERM, BP 10448, F-63000 Clermont-Ferrand, France; elisabeth.noirault@inserm.fr; 5 Laboratoire d'Ingénierie Ostéo-Articulaire et Dentaire-LIOAD, Université de Nantes, UMR 791, INSERM, F-44042 Nantes, France; paul.pilet@univ-nantes.fr

**Keywords:** pomegranate peel extract, nutritional prevention, bone health, cell lines, animal model

## Abstract

The nutritional benefits of pomegranate have attracted great scientific interest. The pomegranate, including the pomegranate peel, has been used worldwide for many years as a fruit with medicinal activity, mostly antioxidant properties. Among chronic diseases, osteoporosis, which is associated with bone remodelling impairment leading to progressive bone loss, could eventually benefit from antioxidant compounds because of the involvement of oxidative stress in the pathogenesis of osteopenia. In this study, with *in vivo* and *ex vivo* experiments, we investigated whether the consumption of pomegranate peel extract (PGPE) could limit the process of osteopenia. We demonstrated that in ovariectomized (OVX) C57BL/6J mice, PGPE consumption was able to significantly prevent the decrease in bone mineral density (−31.9%; *p* < 0.001 *vs.* OVX mice) and bone microarchitecture impairment. Moreover, the exposure of RAW264.7 cells to serum harvested from mice that had been given a PGPE-enriched diet elicited reduced osteoclast differentiation and bone resorption, as shown by the inhibition of the major osteoclast markers. In addition, PGPE appeared to substantially stimulate osteoblastic MC3T3-E1 alkaline phosphatase (ALP) activity at day 7, mineralization at day 21 and the transcription level of osteogenic markers. PGPE may be effective in preventing the bone loss associated with ovariectomy in mice, and offers a promising alternative for the nutritional management of this disease.

## 1. Introduction

Bone is a dynamic organ that is continuously and precisely remodelled by the combined roles of osteoblasts and osteoclasts. Imbalance in bone remodelling is usually caused by a deregulated coupling between the main bone cells due to an increase in osteoclast resorption activity over the bone-formation rate by osteoblasts or by low bone turnover where both formation and resorption are reduced. This process leads to a common adult bone disease, *i.e.*, osteoporosis [1], which is a systemic skeletal disorder characterized by low bone mass and the structural deterioration of the microarchitecture, leading to bone fragility [2]. Many attempts have been made to develop drugs for the prevention and treatment of this disease. Regarding nutritional prevention, despite the traditional focus on calcium and vitamin D, recent preclinical approaches have identified a multitude of nutrients endowed with bone sparing properties [3]. Indeed, it has been shown that polyphenolic compounds such as flavonoids, more precisely, genistein, daidzein, oleuropein, hydroxytyrosol, and others, exert an effect on osteoporosis [4,5,6,7,8,9], inhibit osteoclast differentiation [10,11,12] and stimulate osteoblast formation [13,14,15,16] *in vitro* and *in vivo*.

Among the main dietary sources of such micronutrients, pomegranate (*Punica granatum*, Punicaceae) has been used extensively in the folk medicine of many cultures [17]. While the relationship between pomegranate composition and its biological effects is not clearly elucidated, many recent advances have been made in the effort to discover the mechanisms involved and to identify the molecules responsible for these actions [18,19]. Various parts of the fruit have been shown to exert antioxidant [20,21], anti-inflammatory [22,23], anticarcigogenic [24,25,26], antiatherosclerotic [27,28], hypolipidemic [29,30], antidiabetic [31,32], antiviral, antibacterial, and antifungal activities [33,34,35], on cell lines, in preclinical models and in few human studies [36]. Indeed, the main properties of the pomegranate actually identified thus far are associated with its antioxidant capacity (three times higher than extracts of red wine and tea), and its composition including anthocyanins and tannins [37,38,39]. Actually, it has been reported that reactive oxygen species and low-grade inflammation, both of which contribute to the aging process [40,41,42,43,44,45], are involved in the aetiology of various degenerative diseases, such as osteoporosis [46,47,48,49,50].

The peel of the pomegranate represents almost 26%–30% of the fruit. This part of the fruit has the highest antioxidant capacity (92% of the total antioxidant activity of the fruit [51]) because of its large content of polyphenols such as punicalagin [52], flavonoids (anthocyanins, catechins and other complex flavonoids) and hydrolysable tannins (punicalin, pedunculagin, punicalagin, gallic and ellagic acid) [53]. Pomegranate peel tannins have been widely recognized and used traditionally for their medicinal properties, and several common ailments such as inflammation, diarrhoea, intestinal worms, cough, and infertility have been treated by exploiting pomegranate peel extract [17,54]. Consequently, the exceptional antioxidant and anti-inflammatory potential of pomegranate peel (together with a long history of use in folk medicine), associated with the need to develop new and innovative strategies for the management of osteoporosis, led us to investigate its role in the issue of bone health. In this study, using a pomegranate peel extract rich in tannins, we hypothesized that the consumption of pomegranate peel extract as a dietary supplement could exert a beneficial effect on bone biology. Moreover, using an *in vitro* cell culture model with osteoblasts and osteoclasts, we studied the cellular and molecular mechanisms of action that could be involved, and thanks to a murine model of osteoporosis we attempted to confirm observations at the cellular level.

## 2. Materials and Methods

### 2.1. Pomegranate Peel Extract

**Figure 1:**
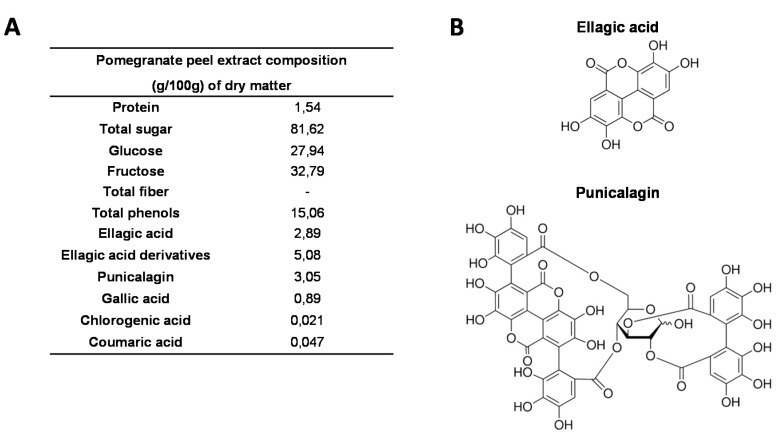
Composition of pomegranate peel extract (PGPE). (**A**) Chemical composition of PGPE (g/100 g dry matter). (**B**) Chemical structure of ellagic acid and punicalagin, the two major polyphenolic compounds of PGPE.

Throughout the study, we used the pomegranate (*Punica granatum* L.) Wonderful cultivar, which is cultured in Israel and was purchased from POMONA (Clermont-Ferrand, France). It has sweet-tart taste, deep purple-red fruits with soft seeds and delicious vinous flavour. For all the *in vitro* and *in vivo* studies, we used the same batch of pomegranates that were stored at 4 °C. A careful sampling was performed for the analyses. Fresh fruits were peeled manually, and the peel was squeezed using a commercial turnmix blender (Philips HR2084, France) to obtain a homogenous puree. Pomegranate peel extract (PGPE) was obtained by hydro-alcoholic (ethanol/water* (mass/mass) 70/30 (*water from pomegranate peel)) extraction from peel puree, with a pomegranate/solvent ratio of 1/15 (w/w), at 90 °C, for 3 h. The extract was then filtrated through a 0.2 μm filter, the ethanol was evaporated and the extract was frozen at −20 °C, directly after preparation and until analysis and diet formulation. The administrated dose of 10 mg polyphenols/kg body weight/day on mice corresponds to a consumption of approximately 93 mg polyphenol/day in human (it is estimated that the spontaneous daily intake of polyphenols is approximately 1 g) [55]. Chemical characterization of PGPE composition was performed by AGROBIO (Rennes, France) and VEGEPOLYS INNOVATION (Angers, France) (see PGPE composition on Figure 1).

### 2.2. Animals Ethics

All animal procedures were approved by the institution’s animal welfare committee (Comité d’Ethique en Matière d’Expérimentation Animale Auvergne: CEMEAA) and were conducted in accordance with the European guidelines for the care and use of laboratory animals (2010-63UE). The animals were housed in the animal facilities of the Human Nutrition Unit at INRA Research Centre [56], Agreement N°: C6334514).

### 2.3. In Vitro Study Design: Serum Production

Fifty 8-week-old female C57BL/6J mice were purchased from JANVIER (St Berthevin, France). After an acclimatization period of one week, the mice were randomly divided into 2 groups (*n* = 25 per group) denoted as the PGPE and control (CT) groups. Both groups were fed a control diet (AIN-93G). PGPE (50 mg polyphenol/kg body weight/day ellagic acid equivalent) was given to each animal in the PGPE group by forced oral administration, while the control group received the same amount of physiological saline, using the same procedure. After 10 days, venous blood from all the animals was collected under anaesthesia and centrifuged (3000 rpm for 5 min at room temperature). Serum supernatant was subsequently harvested. Then, serum from the same animal group was pooled, filtered at 45 μm, divided into aliquots and stored at −80 °C until use in the cell culture medium.

#### 2.3.1. Cell Lines and Culture Conditions

Murine MC3T3-E1 osteoblast cells (ATCC, Washington, DC, USA) were seeded on collagen-1-coated plates (BD Biosciences, Bedford, MA, USA) at a density of 3 × 10^4^ cells/cm^2^. The cells were maintained in α-minimal essential medium (α-MEM; GIBCO, Paisley, UK) supplemented with 1% penicillin/streptomycin (GIBCO, Paisley, UK) and 10% heat-inactivated fetal bovine serum (FBS, Lonza, Levallois-Perret, France) (minimal medium). At 80% confluence, the cells were exposed to different conditions: minimal medium (C−) or minimal medium containing 50 μg/mL ascorbic acid and 10 mM β-glycerophosphate (C+; differentiation medium), in the presence of 10% heat-inactivated FBS (FBS) or 7.5% heat-inactivated FBS + 2.5% serum from mice that received physiological serum (Control) or pomegranate peel extract (PGPE), for 21 days.

In addition, RAW264.7, a murine monocytic cell line (ATCC, Washington, DC, USA), was used as an osteoclast model. The cells were seeded at a density of 1 × 10^4^ cell/cm^2^ and maintained in *α*-minimal essential medium (*α*-MEM; GIBCO, Paisley, UK) supplemented with 1% penicillin/streptomycin (GIBCO, Paisley, UK) and 10% heat-inactivated fetal bovine serum (FBS) (minimal medium). At 80% confluence, the cells were exposed to different conditions: minimal medium (C−) or minimal medium containing 50 ng/mL receptor activator of nuclear factor-kappa B ligand (RANK-L) (R&D Systems) (C+; differentiation medium), in the presence of 10% heat-inactivated FBS (FBS) or 7.5% heat-inactivated FBS + 2.5% serum from mice that received physiological serum (Control) or pomegranate peel extract (PGPE) for 4 days.

The replacement proportion of FBS by mice serum in these studies was determined by preliminary studies employing RAW264.7 and MC3T3-E1 cells to ensure that no modification of cell differentiation (cell viability, proliferation, specific activity, expression of major makers of cell type) was induced.

Both cell types were cultured at 37 °C in a humidified atmosphere of 5% CO_2_ in air. The medium was replaced every 2 days. All experiments were repeated at least 3 times and reproduced 2 times.

#### 2.3.2. Cell Proliferation

RAW264.7 and MC3T3-E1 cells were seeded in a 96-well plate at a density of 3.5 × 10^3^ cells per well and then cultured for 48 h or 7 days with 2% serum (2% FBS or 1.25% FBS + 0.75% CT or PGPE mice serum). Cell viability was determined by an XTT-based method using Cell Proliferation Kit II (Sigma-Aldrich, St. Louis, MO, USA) according to the manufacturer’s recommendations. The OD was determined at 450 nm.

#### 2.3.3. Alkaline Phosphatase (ALP) Activity Measurement

The enzymatic activity of ALP was measured in osteoblasts at days 0, 2, 7 and 14, according to previously published methods [57], adapted to our experimental conditions. Briefly, the osteoblast cultures were rinsed twice with PBS (Sigma-Aldrich, 38297 Saint-Quentin Fallavier, France) prior to freezing at −20 °C. The cells were then lysed by freeze–thaw cycles and homogenized in diethanolamine/magnesium chloride hexahydrate buffer (pH 9.8; Sigma-Aldrich). The cell lysates (10 μL) were added to 200 μL of p-nitrophenyl phosphate solution (6 mg/mL) (Sigma-Aldrich, St. Louis, MO, USA). Absorbance was assessed at 405 nm, 30 °C, every 150 s for 30 min, using an ELX808 microplate reader (BioTek Instruments Inc, Winooski, VT, USA). Protein measurement was performed using a BioRad protein assay (BioRad, Munich, Germany). ALP activity was expressed as micromoles of *p*-nitrophenol per hour per milligram of protein [58].

#### 2.3.4. Tartrate-Resistant Acid Phosphatase (TRAP) Activity Measurement

TRAP activity was assayed according to the standard method using *p*-nitrophenyl phosphate as a substrate [59]. The medium was removed from osteoclasts cultured in 24-well plates after 3 days, and cell lysates were prepared using NP40 lysis buffer. The samples were incubated in assay buffer (125 mM sodium acetate buffer (pH 5.2), 100 mM *p*-nitrophenyl phosphate (Sigma-Aldrich, St. Louis, MO, USA), and 1 mM L(+) sodium tartrate). The production of *p*-nitrophenol was determined at 405 nm, 37 °C and expressed as the mean OD per minute per milligram protein.

#### 2.3.5. Mineralization

Osteoblasts seeded in 12-well plates were cultured for 21 days in minimal medium containing 50 μg/mL ascorbic acid and 10 mM β-glycerophosphate to investigate bone nodule formation. Extracellular matrix calcium deposits were stained using Alizarin red dye, as previously described [58]. Osteoblasts were fixed with ice-cold 70% (*v*/*v*) ethanol and then stained with Alizarin Red S (40 mM) (Sigma-Aldrich, St. Louis, MO, USA) for 30 min. Mineralization was evaluated by the light microscopy and image processing software ImageJ (National Institutes of Health, Bethesda, MD, USA).

### 2.4. In Vivo Study Design

Thirty 8-week-old female C57BL/6J mice were purchased from JANVIER (St Berthevin, France). After an acclimatization period of one week, the mice were randomly divided into 3 groups (*n* = 10 per group). Two groups were surgically ovariectomized (OVX), under ketamine/xylazine anaesthesia, while the other animals were sham operated (SH). Paracetamol was added to the water for 24 h post-surgery for pain limitation [60,61].

Control mice (SH Control and OVX Control) were fed a standard AIN-93G diet, while the last group (OVX PGPE) received the standard diet enriched with 2 g/kg of PGPE (*i.e.*, 10 mg polyphenols (ellagic acid equivalents)/kg body weight/day). The diets were purchased from SAFE (Scientific Animal Food and Engineering, Augy, France).

The animals were housed in a controlled environment (12:12 h light-dark cycle, at 20–22 °C, with 50%–60% relative humidity, 1 mouse per plastic cage), with free access to water. Body weight was measured every two days during the study period. Body composition was assessed at the beginning and at the end of the study, using a QMR (Quantitative magnetic resonance) EchoMRI-900™ system (EchoMRI LLC, Houston, TX, USA), without any anaesthesia or sedation. After the 30-day treatment, the mice were sacrificed. The liver, spleen and uterus were weighted. Femurs and tibias were harvested and stored at −80 °C prior to investigation.

#### 2.4.1. Bone Mineral Density (BMD) Analysis

Bone morphological analysis was performed using an eXplore CT 120 scanner (GE Healthcare, Little Chalfont, United Kingdom). After the removal of soft tissues, the left femurs were placed in PBS buffer with 10% formaldehyde at 4 °C for one week and scanned. Acquisition consisted of 360 views acquired in 1 soft tissues, the left femurs were placed in PBS buffer with 1 millisecond exposure/view and X-ray tube settings of 100 kV and 50 mA. CT images were reconstructed using a modified conebeam algorithm with an isotropic voxel of 0.045 x 0.045 x 0.045 mm^3^. CT scans were analysed using MicroViewH version 2.3 software (General Electric Healthcare Bio-Sciences, Pittsburgh, PA, USA). A hydroxyapatite calibration phantom (SB3, Gamex RMI, WI, USA) was used to convert grey-scale levels to hydroxyapatite density values. The trabecular bone of the distal femur was selected for bone mineral density and bone volume fraction (BVF = Bone Volume/Total volume) analyses by fitting a cylindrical region (*r* = 0.7 mm) of interest in the center of the femur, starting 0.1 mm proximally from the growth plate and extending a further 0.32 mm in the proximal direction. Bone mineral density was estimated as the mean converted grey-scale level within the region of interest.

#### 2.4.2. Bone Micro-Architecture Analysis

To perform a measurement, the specimen was mounted on a turntable that could be shifted automatically in the axial direction (angular step: 0.675 /reconstruction angular range: 186.30). An aluminium filter (0.5 mm thick) was placed between the X-ray source and the sample. The micro-architecture (secondary spongiosa) was analysed using X-ray radiation provided by a micro-CT SkyScan 1072 (BRUKERMICROCT, Kontich, Belgium). Pictures of 1024 × 1024 pixels were obtained using 37 kV and 215 mA. According to the camera settings, the final pixels measured 5.664 mm, leading to a voxel of 1.817 × 1027 mm^3^. The calculation of histomorphometric parameters was performed using the CTAn H version 1.11 (BRUKER MICROCT, Kontich, Belgium), and NreconH software version 1.6.1.7 [62] (BRUKER MICROCT, Kontich, Belgium).

### 2.5. Taqman Low Density Arrays (TLDA)

For each experimental group, four sets of extractions were performed with two tibias pooled in each. Frozen bones were ground in liquid nitrogen to obtain a fine powder. Then, total RNA was extracted according to the manufacturer's instructions from either bone powder for the *in vivo* experiment or cell culture, using Trizol reagent (Invitrogen, Life Technologies, Saint Aubin, France). RNA was converted to cDNA using the high capacity cDNA reverse transcription (RT) kit (Applied Biosystems). The resulting cDNA was used for TaqMan^®^ low-density arrays (TLDAs) (Applied Biosystems 7900HT real-time PCR system). Relative expression values were calculated using the comparative threshold cycle (2^−ΔΔ^CT) according to the Data Assist software (Applied Biosystems, Life Technologies, Saint Aubin, France). 18S, GAPDH and actin served as housekeeping genes.

### 2.6. Statistical Analysis

Results are expressed in the form of mean with standard error (SEM). All the data were analysed using XLSTAT Pro software [63] (Addinsoft, 40 rue Damrémont, Paris, France). *In vitro* data were analysed using a two-way analysis of variance (ANOVA) to test for differences among groups. If the result was found to be significant (*p* < 0.05), the fisher multiple comparison test was then performed to determine the specific difference between means. TLDA data and *in vivo* data were analysed using non-parametric Kruskal–Wallis one-way analysis of variance, which allows testing for differences among groups. If the result was found to be significant (*p* < 0.05), the Mann-Whitney U test was performed to determine the specific difference between means.

## 3. Results

To determine the accurate potential effect of pomegranate peel extract (PGPE) on bone metabolism, we first developed an *in vitro* model relevant to nutritional conditions. This model allowed us to consider plasma modifications elicited by PGPE consumption (including both production of metabolites from the pomegranate polyphenols and any systemic effects of those molecules) and to study the impact of pomegranate on bone cell cultures *in vitro*. For that purpose, serum from mice that had been given PGPE or physiological saline (control) was introduced into the medium of bone cell cultures (in which 25% of FBS was replaced by mouse serum: 7.5% heat-inactivated FBS + 2.5% serum from mice).

### 3.1. nPGPE Promoted Osteoblast Differentiation

#### 3.1.1. Cell Viability

**Figure 2:**
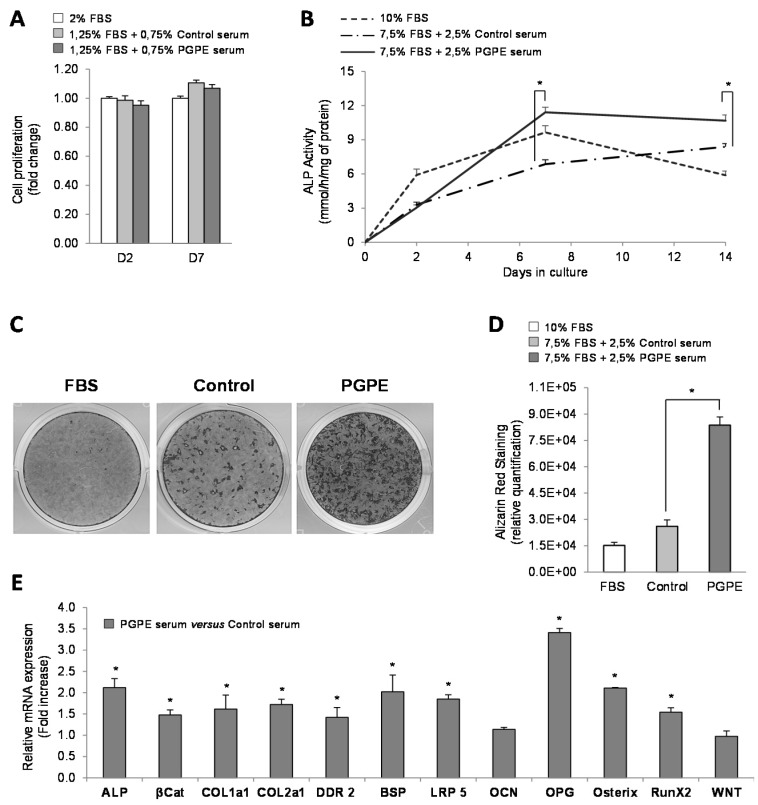
Osteoblast viability, differentiation and transcription in culture, after exposure to serum harvested from mice raised either under control conditions or treated with pomegranate peel extract. * *p* < 0.05 PGPE *versus* Control condition. (**A**) Cell proliferation test performed on MC3T3-E1 cells cultured in minimal medium (2% Fetal Bovine Serum, FBS), supplemented with serum from mice given either physiological serum (1.25% FBS + 0.75 Control mice serum) or pomegranate peel extract, PGPE (1.25% FBS + 0.75 PGPE mice serum), after 2 and 7 days of treatment. Results are expressed as mean ± standard error of the mean (SEM) (*n* = 6). (**B**) Alkaline phosphatase, ALP activity of MC3T3-E1 cells cultured in optimized medium with 10% FBS or with 7.5% FBS + 2.5% serum from mice fed either physiological serum (Control) or pomegranate peel extract (PGPE), after 0, 2, 7 and 14 days of treatment. Results are expressed as mean ± SEM (*n* = 8). (**C**,**D**) Mineralized nodules stained with alizarin red S on Day 21 in MC3T3-E1 cells cultured in optimized (C+) medium with 10% FBS or with 7.5% FBS + 2.5% serum from mice exposed to either physiological serum (Control) or pomegranate peel extract (PGPE). (**C**) Representative image of alizarin red staining for each condition. (**D**) Relative quantification of alizarin red staining with picture analyser software ImageJ. Results are presented as mean ± standard deviation, SD (*n* = 6). (**E**) Transcriptomic analysis of the main osteoblast markers in MC3T3-E1 cells cultured in optimized medium after 7 days of treatment with serum from mice exposed to either physiological serum (Control) or pomegranate peel extract (PGPE). Transcriptomic analysis of MC3T3-E1 mRNA levels determined by Taqman Low-Density Arrays are presented as fold change compared to the Control condition (fold change = 1) as mean ± SD (*n* = 6). Genes: ALP: alkaline phosphatase; βCat: β-catenin；Coll1a1: collagen 1a1; Coll2a1: collagen 2a1; DDR2: discoidin domain receptor 2; BSP: bone sialoprotein; Lrp5; OCN: osteocalcin; OPG: osteoprotegerin; osteri RunX2 and Wnt.

To insure that mouse sera (harvested from mice fed either a standard diet (Control) or the control diet enriched with PGPE) were not deleterious for MC3T3-E1 cells, a cell viability test was performed. Incubation with serum from PGPE animals did not modify the proliferation of the MC3T3-E1 cells, on days 2 and 7, in comparison with cells cultured with serum from CT mice (Figure 2A).

#### 3.1.2. Effect of PGPE on ALP Activity and Mineralization of MC3T3-E1 Cells

The kinetic measurement of ALP activity in cells treated with mice serum raised in either Control or PGPE conditions on days 2, 7 and 14 was first assessed to investigate the effect of the tested diets on osteoblast differentiation. With regard to the Control condition (FBS), as expected, the osteoblasts treated with optimized medium (C+) exhibited higher ALP activity compared to osteoblasts exposed to a minimal medium (C−), as of day 2 (+72%), day 7 (+374%; *p* < 0.01) and day 14 (+139%) (data not shown). The cells that had been exposed to PGPE mice serum exhibited higher values compared to the Control condition (+46% at day 7 and +16% at day 14; *p* < 0.05). (Figure 2B).

In addition to this ALP activity and to target the effect of PGPE on bone mineralization, we analysed calcium deposition by MC3T3-E1 cells under differentiated conditions (C+) and when treated with either PGPE mouse serum or Control mouse serum for 21 days. As shown in Figure 2C, and as expected, the osteoblasts treated with PGPE mouse serum were able to produce many more calcium nodules after 21 days of culture than osteoblasts treated with Control mouse serum (Figure 2D).

#### 3.1.3. Effect of PGPE on transcriptional activity of MC3T3-E1 cells

To better understand the effect of PGPE on osteoblasts, transcriptional analyses of major differentiation and transcription factors were performed. Due to the large number of targets, only the most relevant and significant ones are presented. The expression level of mRNA extracted from MC3T3-E1 cells after 7 days of culture with serum from PGPE mice was compared to the expression level of mRNA from cells treated with serum from Control mice at the same culture time (Fold increase value = 1). As shown in Figure 2E, PGPE led to a significant (*p* < 0.05) increase in the expression of the major osteogenic markers such as ALP (2.12-fold), collagen 1a1 and 2a1 (Coll1a1 and Coll2a1) (1.62-fold and 1.72-fold respectively), bone sialoprotein (BSP) (2.02-fold), osteocalcin (1.14-fold; not significant) and osteoprotegerin (OPG) (3.41-fold). In addition, discoidin domain receptor 2 (DDR2), a cell surface receptor that plays critical roles in the collagen control of osteoblast differentiation, was up-regulated by PGPE (1.42-fold; *p* < 0.05). Two members of the Wnt/β-catenin signalling pathway that regulate osteoblast differentiation and bone formation were also up-regulated by PGPE: β-catenin (1.47-fold; *p* < 0.05) and LRP5 (1.85-fold; *p* < 0.05), but not Wnt (0.97 fold). Finally, PGPE stimulated the expression of the two master osteoblastic transcription factors: osterix and runX2 (2.11- and 1.54-fold respectively; *p* < 0.05).

### 3.2. PGPE Did Not Affect Osteoclast Activity but Impaired Transcription

#### 3.2.1. Cell Viability

**Figure 3:**
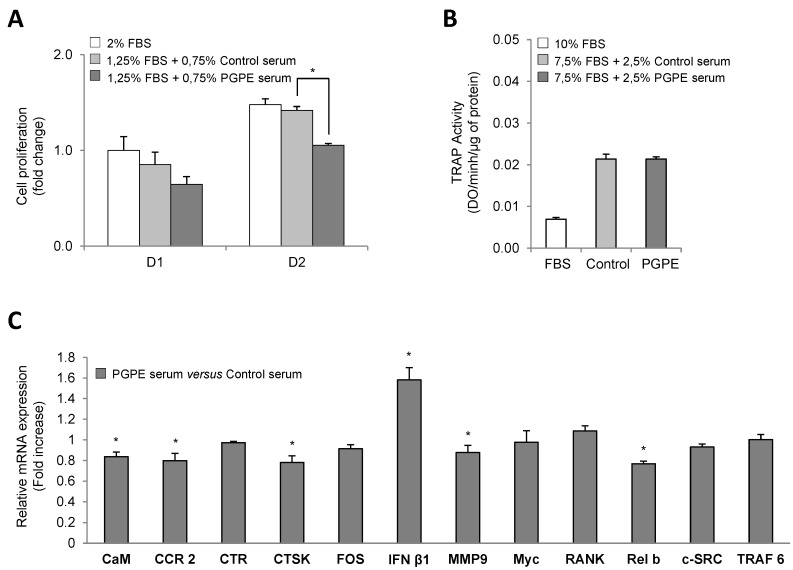
Osteoclast viability, differentiation and transcription in culture after exposure to serum harvested from mice either raised under control conditions or treated with pomegranate peel extract. * *p* < 0.05 PGPE *versus* Control condition. (**A**) Cell proliferation test performed on RAW264.7 cell line cultured in minimal medium (2% Fetal Bovine Serum, FBS), supplemented with serum from mice exposed to either physiological serum (1.25% FBS + 0.75% Control mice serum) or pomegranate peel extract, PGPE (1.25% FBS + 0.75 PGPE mice serum) after 1 and 2 days of treatment. Results are expressed as mean ± standard error of the mean (SEM) (*n* = 6). (**B**) Tartrate resistant acid phosphatase, TRAP activity of RAW264.7 cells cultured in optimized medium with 10% FBS or with 7.5% FBS + 2.5% mice serum from animals fed either physiological serum (Control) or pomegranate peel extract (PGPE), after 3 days of treatment. Results are expressed as mean ± SEM (*n* = 8). (**C**) Transcriptomic analysis of the main osteoclast markers in RAW264.7 cells cultured in optimized medium after 2 days of treatment with serum from mice exposed to either physiological serum (Control) or pomegranate peel extract (PGPE). Transcriptomic analysis of the Raw264.7 mRNA levels determined by Taqman Low density Arrays are presented as fold change compared to Control condition (fold change = 1) as mean ± standard deviation (SD) (*n* = 6). Gene: CaM: calmodulin; CCR2: chemokine receptor 2; CTR: calcitonin receptor; CTSK: cathepsin K; FOS; IFN β1: interferon β1; MMP9: metalloproteinase 9; Myc, RANK: receptor activator of nuclear factor-κB; Relb; c-SRC: and TRAF6.

With regard to the osteoclast model (RAW264.7 cells), proliferation was significantly increased at day 2 in all conditions, in comparison to day 1. However, mouse serum induced a slight decrease in osteoclast proliferation on the first culture day compared to what was observed in the FBS condition. No significant difference was noticed between the PGPE and Control serum conditions (−36% and −21% respectively; *p* < 0.5) (Figure 3A). Then, on day 2, we observed that in the PGPE condition, osteoclast proliferation was reduced compared to the Control mouse serum condition (−26%, *p* < 0.01).

#### 3.2.2. Effect of PGPE on TRAP Activity of RAW264.7 Cells

We assessed the TRAP activity in RAW264.7 cells after three days of culture to determine whether serum from PGPE mice was able to reduce osteoclast differentiation. As described in Figure 3B, exposure to mouse serum was associated with an increase of such a parameter. However, PGPE did not elicit any change compared to observations using serum from control mice.

#### 3.2.3. Effect of PGPE on Transcriptional Activity of RAW264.7 Cells

To further investigate the effect of PGPE on bone resorption, the expression levels of specific differentiation markers and transcription factors were assessed. A TLDA analysis was therefore performed on mRNA extracted from RAW264.7 cells treated with mouse serum (Control or PGPE) under the differentiated condition (C+) after three days of culture. The expression of the most relevant targets is shown in Figure 3C. The exposure to serum from PGPE mice was compared to the results of Control treated cells (Fold increase value = 1). The TLDA data indicate that the exposure of RAW264.7 differentiated cells to serum from PGPE mice was associated with a slight, but significant (*p* < 0.05), decrease in the mRNA levels of many crucial osteoclast-specific markers, such as calmodulin (CaM) (0.84-fold), chemokine receptor 2 (CCR2) (0.80-fold), cathepsin K (CTSK) (0.78-fold) and metalloproteinase 9 (MMP9) (0.88-fold) (Figure 3C). Consistently with this effect, down-regulated expression of a downstream NF-κB subunit, RelB, was observed in the PGPE condition (0.77-fold; *p* < 0.05). Moreover, the expression of interferon β1 (INFβ1), an osteoclastogenesis inhibitor, was induced (1.58-fold; *p* < 0.05) in the PGPE condition.

### 3.3. Confirmation of Cell Culture Results Using a Physiological in Vivo Approach

These *ex vivo* results were completed by an *in vivo* investigation of the possible effect of pomegranate using a well-described model of osteoporosis induction by ovariectomy on mice.

#### 3.3.1. Validation of the Animal Model

During this study, the mean body and uterine weights, as well as the lean and fat mass, were measured to ensure that our model conformed to published data. In Table 1, we observed that anthropometric parameters (total weight, fat and lean mass) increased in all the experimental groups throughout the experiment. No significant difference was observed between groups, SH Control *vs.* OVX Control or OVX PGPE *vs.* OVX Control. As expected, ovariectomy significantly induced marked uterine atrophy in the OVX Control group (Table 1). This effect on the uterus was not prevented by pomegranate peel extract consumption.

**Table 1:** Effect of ovariectomy and pomegranate peel extract consumption for 30 days on physiological parameters in mice.

	Sham	Ovariectomized	
	Normal Diet	Normal Diet	PGPE Diet
	(SH)	(OVX)	(OVX+PGPE)
Initial body weight (g)	18.9 ± 0.6	19.4 ± 0.6	19.0 ± 0.5
Initial lean mass (% body weight)	87.6 ± 3.8	87.1 ± 1.2	87.1 ± 0.9
Initial fat mass (% body weight)	6.6 ± 2.1	7.9 ± 1.3	8.0 ± 1.2
Final body weight (g)	19.3 ± 0.8	20.3 ± 1.2	19.9 ± 0.9
Final lean mass (% body weight)	88.9 ± 7.3	92.3 ± 3.0	91.6 ± 2.7
Final fat mass (% body weight)	9.8 ± 2.2	9.3 ± 1.6	9.0 ± 2.0
Uterine weight (mg)	84.4 ± 3.2	23.1 ± 2.8 #	23.7 ± 2.8

Mice were sham-operated, SH or ovariectomized, OVX and fed standard diet AIN-93 (Control) or standard diet enriched with 2g/kg of pomegranate peel extract, PGPE representing a dose of 10 mg polyphenols/kg body weight/day (ellagic acid equivalent) for 30 days. Each group contained 10 mice. Values are presented as means ± standard error of the mean, SEM. # *p* < 0.005 OVX Control versus SH Control group.

**Figure 4:**
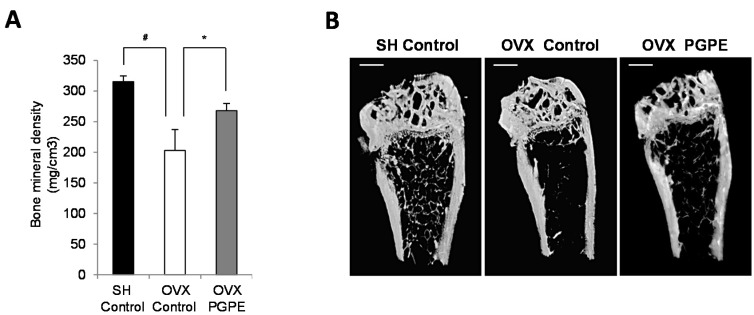
Effect of ovariectomy and pomegranate peel extract consumption for 30 days on bone parameters in mice. (**A**) Bone mineral density analysis of left femur by X-ray radiation micro-CT. Mice were sham-operated, SH or ovariectomized, OVX and fed standard diet AIN-93 (Control) or standard diet enriched with 2g/kg of pomegranate peel extract, PGPE representing a dose of 10 mg polyphenols/kg body weight/day (ellagic acid equivalent) for 30 days. Each group contained 10 mice. Results are presented as means ± standard error of the mean, SEM. # *p* < 0.005 OVX Control versus SH Control group. * *p* < 0.05 OVX PGPE *versus* OVX Control. (**B**) Representative microCT images of the distal left femur for each group: SH Control, OVX Control and OVX PGPE. Scale bars, 1 mm.

#### 3.3.2. PGPE Consumption Improved Bone Mineral Density in Ovariectomized Mice

Bone morphological parameters using micro-CT analysis were first assessed to evaluate the respective effects of ovariectomy and PGPE consumption on mice. We confirmed the acknowledged effect of oestrogen deficiency on femoral BMD by comparing the OVX Control animals and the SH Control group. Indeed, the OVX Control mice had a significant lower BMD (*p* < 0.001) than the SH Control mice (−35.6%) (Figure 4A). PGPE consumption was associated with the prevention of such a bone decrease in OVX animals (+31.9%; *p* < 0.001 compared to the OVX Control animals).

#### 3.3.3. PGPE Preserved Bone Microarchitecture in Ovariectomized Mice

We also observed a significant effect on the trabecular bone microarchitecture of the distal femur, as shown in Figure 4B. Again, as expected, bone microarchitecture was significantly impaired by ovariectomy, with an increase in trabecular separation (TbSP +26.1%; *p* < 0.001) as well as total porosity (Po tot +3.5%; *p* < 0.01) in comparison with the SH Control group (Table 2). Significant decreases in trabecular number (TbN: −25.0%; *p* < 0.05), bone per cent volume (BV/TV: −23.6%; *p* < 0.01), connectivity density (Conn Dn: −19.7%; *p* < 0.01) and bone surface density (BS/TV: −23.9%; *p* < 0.05) were also observed. In addition, the consumption of PGPE exhibited a protective effect on bone microarchitecture, as PGPE consumption improved significantly (*p* < 0.01): BV/TV (+23.1%), TbN (+22.6%), structure model index (SMI: −7.3%), Po tot (−2.52%) and BS/TV (+17.1%) compared to the values measured in the OVX Control animals.

**Table 2:** Effect of ovariectomy and pomegranate peel extract consumption for 30 days on bone micro-architectural parameters in mice.

	Sham	Ovariectomized	
	Normal Diet	Normal Diet	PGPE Diet
	(SH)	(OVX)	(OVX+PGPE)
BV/TV (%)	12.7 ± 0.9	9.7 ± 0.6 #	11.9 ± 0.76 *
TbTh (mm)	0.1 ± 0.0	0.1 ± 0.0	0.1 ± 0.0
TbN (mm^−1^)	2.0 ± 0.1	1.5 ± 0.1 #	1.8 ± 0.07 *
TbSP (mm)	0.2 ± 0.0	0.3 ± 9.97E-03 #	0.3 ± 0.0
SMI	2.5 ± 0.1	2.6 ± 0.1	2.4 ± 0.04 *
Conn Dn (mm^−3^)	278.4 ± 26.5	223.5 ± 8.8	224.3 ± 12.7
Tb Pf (mm^−1^)	25.5 ± 1.1	27.7 ± 1.0	23.2 ± 1.2
Po tot (%)	87.3 ± 1.0	90.3 ± 0.61 #	88.1 ± 0.76 *
BS/TV (mm^−1^)	8.9 ± 0.5	6.7 ± 0.32 #	7.9 ± 0.31 *

Mice were sham-operated, SH or ovariectomized, OVX and fed standard diet AIN-93 (Control) or standard diet enriched with 2g/kg of pomegranate peel extract, PGPE representing a dose of 10 mg polyphenols/kg body weight/day (ellagic acid equivalent) for 30 days. Each group contained 10 mice. Values represent means ± standard error of the mean, SEM. # *p* < 0.005 OVX Control *versus* SH Control group. * *p* < 0.05 OVX PGPE *versus* OVX Control. Abbreviations: BV/TV: Per cent bone volume; TbTh: trabecular thickness; TbN: trabecular number; TbSP: trabecular spacing; SMI: structure model index; ConnDn: connectivity density; Tb Pf: trabecular pattern factor; Po tot: total porosity; BS/TV: bone surface density.

#### 3.3.4. Pomegranate Intake Was Associated with an Improved Expression Profile of Specific Bone Markers

**Figure 5:**
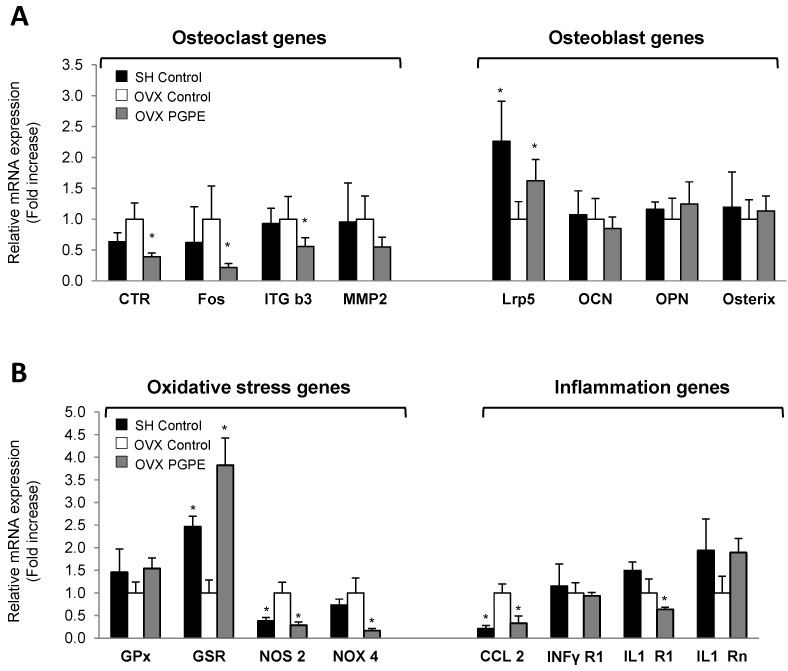
Expression profile analysis of bone (**A**), oxidative stress and inflammation (**B**) markers in femoral bone from mice fed the control diet (Sham-operated, SH Control. Ovariectomized, OVX Control) or exposed to pomegranate peel extract (OVX PGPE, diet containing 2 g/kg of PGPE), for 30 days. Transcriptomic analysis of bone tissue mRNA levels determined by Taqman Low density Arrays are presented as fold change compared to OVX Control group (fold change = 1). Results are expressed as mean ± standard deviation, SD (*n* = 8). * *p* < 0.05. (**A**) Osteoclast genes: CTR: calcitonin receptor; Fos; ITG b3: integrin β3; MMP2: metalloproteinase 2. Osteoblast genes: Lrp5; OCN: osteocalcin; OPN: osteopontin; RunX2. (**B**) Oxidative stress genes: GPx: glutathione peroxidase; GSR: glutathione reductase; NOS2: nitric oxide synthase 2; NOX4: NADPH oxidase 4. Inflammation genes: CCL2: Chemokine (C-C motif) ligand 2; INFγR1: interferon gamma receptor 1; IL1-R1: interleukin 1 receptor 1; IL1-Rn: interleukin 1 receptor antagonist.

To elucidate the possible molecular mechanisms involved in this effect on bone morphology and to confirm the *in vitro* findings, we performed a transcriptomic analysis on bone tissue samples using Taqman Low Density Arrays (TLDA, Applied Biosystems, Life Technologies, Saint Aubin, France). Because of the large number of genes modulated, only the most important bone markers are presented. Data on the OVX PGPE group were compared to the data measured for the OVX Control group. The TLDA data on osteoclasts are shown in Figure 5A. Accordingly, PGPE consumption significantly (*p* < 0.05) down-regulated the mRNA levels of some of the major osteoclast-specific markers: define (0.39 fold) and integrin β3 (ITG b3: 0.56 fold). Fos, a crucial transcription factor for osteoclast differentiation, was also inhibited in the PGPE group (0.22 fold; *p* < 0.05). Finally, we observed a trend toward down-regulated expression in the OVX PGPE group of a key enzyme for osteoclast activity involved in collagen degradation: the metallo-proteinase 2 (MMP2) (PGt: 0.55; not significant). In addition, regarding the expression of osteoblast-specific markers, the TLDA data presented in Figure 5A show that PGPE consumption by ovariectomized mice was able to significantly improve the expression of a co-receptor implied in Wnt/β-catenin signalling in the osteoblast, LRP5 (1.62 fold; *p* < 0.05), in comparison with the OVX Control group. However, the other osteoblast markers investigated were not significantly modulated by dietary PGPE.

#### 3.3.5. PGPE Enhanced Bone Inflammatory and Oxidative Status Marker Expression in Ovariectomized Mice

To obtain further conclusions regarding the potent bone-sparing effect of pomegranate consumption, we investigated the expression of major inflammation and oxidation markers, which are known to exacerbate bone resorption. We therefore analysed the transcription of some inflammatory and oxidation markers in bone tissue samples using Taqman Low Density Arrays (TLDA, Applied Biosystems, Life Technologies, Saint Aubin, France). Regarding the expression of oxidation markers in the bone microenvironment, dietary PGPE enhanced bone oxidative stress in ovariectomized mice compared to mice given the standard diet, as shown in Figure 5B. PGPE consumption up-regulated the expressions of antioxidant enzymes (GPx (1.54-fold; trend) and GSR (3.82-fold; *p* < 0.05)), while the expression of the ROS synthesis enzyme NOS2 was down-regulated (0.16-fold; *p* < 0.001). In addition, PGPE consumption by ovariectomized mice was able to prevent the instigation of a low-grade inflammatory status compared to OVX Control animals, through the down-regulated expression of CCL2 (0.33-fold; *p* < 0.05) and IL1-R1 (0.64-fold; *p* < 0.001) and the up-regulated expression of IL1-Rn (1.89-fold; *p* < 0.001), again supporting our previous results.

## 4. Discussion

Taken together, these results outline the potential effect of pomegranate peel extract consumption on bone health. Indeed, using an *ex vivo* investigation that makes it possible to address the overall physiological conditions (by considering the specific metabolism of pomegranate peel extract polyphenols and the possible systemic effects of the consumption of such ingredients) coupled with a well-described preclinical model, we demonstrated that dietary PGPE could improve bone mass and microarchitecture with transcriptional changes in bone tissue during osteoporosis establishment.

Our PGPE is composed of free glucose and fructose (~80%) and polyphenols (~15%) comprising mostly ellagitannins: ellagic acid (~2.9%), derivatives (~5.1%) and punicalagin (~3.1%). In the body, the native ellagitannin forms of PGPE are converted to ellagic acid through a (non-enzymatic) reaction due to acid hydrolysis, which occurs in the caecum at a pH of 8. Punicalagin catabolism leads to the release of ellagic acid, which is largely absorbed and converted by the bacterial microflora in the intestinal lumen to produce the main metabolites of punicalagin, urolithins (mainly forms A and B in humans). While ellagitannins are not found in either the plasma or the urine after the ingestion of pomegranate, their metabolites (ellagic acid and urolithins) are present [23,64,65]. In our studies, the maximal doses of PGPE administered were 10 (*in vivo* study) or 50 (*in vitro* study) mg ellagic acid equivalents/kg body weight/day. These doses were safe, as it has been reported that the oral administration of 600 mg/kg/day of pomegranate extract to rats, corresponding to a dose of 180 mg punicalagin/kg/day for 90 days, was devoid of any observed adverse effect level (NOAEL) [66]. Thus, using serum from mice fed with PGPE or physiological serum (Control) on *in vitro* bone cell culture, we attempted to reproduce the bone nutritive microenvironment induced by PGPE nutritional supplementation in the animals. Indeed, in physiological conditions, bone cells are never in direct contact with the native form of those molecules.

Using our *ex vivo* MC3T3-E1 cell line model, we found that PGPE significantly stimulated osteoblastogenesis, as demonstrated by increased ALP activity and calcium nodule formation. The transcriptional analysis of major osteoblast lineage markers was consistent with those two markers of osteoblast activity: ALP, BSP, Coll1a1, Coll2a1, DDR2, OCN, OPG were all significantly up-regulated by PGPE. Similarly, an increase in the expression of the two main osteoblastic transcription factors, osterix and runx2, allowed us to hypothesize that transcriptional changes might contribute to the improved osteoblast function [67,68]. Previous works conducted using MC3T3-E1 cells have suggested an osteogenic role of a pomegranate ethanolic extract [20] or pomegranate concentrate powder extract [69] underlined by increased ALP activity, even though the authors did not consider the particular metabolism of pomegranate micronutrients.

We also investigated, for the first time, the effects of PGPE on bone cell resorption based on an *in vitro* concept using RAW264.7. We found that serum from PGPE mice was able to inhibit RANKL-induced osteoclast differentiation, as shown by the down-regulation of the expression of specific osteoclast markers (calmodulin, CCR2, calcitonin receptor, cathepsin K, MMP-9) [70]. We observed a consistent decreased expression of relB, the downstream NF-κB subunit responsible for osteoclast differentiation [71]. In addition, the expression of interferon β1, an inhibitor of osteoclastogenesis [72], was up-regulated, thus providing a potential explanation for the PGPE-related inhibition of osteoclastogenesis. Consistently with our data, an *in vitro* anti-resorptive activity of pomegranate pericarp extract has also been suggested, as it was shown to inhibit the receptor activator of nuclear factor-κB ligand (RANKL) expression in MG-63 human osteosarcoma cells [73]. To date, except for the work conducted with ellagitannin (furosin, extracted from Euphorbia helioscopia) on osteoclasts [74], showing the suppression of RANKL-induced osteoclast differentiation and function through the inhibition of MAP kinase activation, no data have targeted the role of pomegranate polyphenols on RAW264.7 osteoclasts.

In agreement with those results, *in vivo* investigation here also demonstrated that PGPE consumption was associated with the decrease of pro-resorption marker expression using Taqman Low density Arrays.

In addition to the direct effects on bone demonstrated by our *in vitro* data, PGPE could modulate bone physiology through other mechanisms. Native ellagitannin forms of PGPE are hydrolysed by intestinal microflora into punicalagins and ellagic acid, which act as prebiotics. These molecules might contribute to the positive effect on bone [75,76]. They are known to enhance the growth of bifidobacteria and lactobacilli in the intestine, leading to increased production of short chain fatty acids (SCFAs) [77]. SCFAs contribute directly to the enhancement of Ca absorption via a cation exchange mechanism (increased exchange of cellular H^+^ for luminal Ca^2+^) [78,79]. The extract of pomegranate pericarp could act as selective oestrogen receptor modulators (SERMs ) on bone as well, as it has an antioestrogenic effect in the mammary gland, without compromising the beneficial effects of oestrogen in the cardiovascular and skeletal system [80]. This SERM-like effect could be attributed to ellagic acid. *In vivo*, this compound prevents bone loss after tooth extraction in normal [81] or diabetic rats [82] with an increase of ALP expression. In ovariectomized (OVX) rats, an extract of Punica granatum peel has been shown to induce a simultaneous, dose-dependent increase in femoral BMD and uterine weight, suggesting a potent oestrogenic activity of the ellagic acid present in the extract [83]. These effects were equivalent to the effects of Tamoxifen in increasing femoral mineral content and osteoblast number [84]. Moreover, OVX rats fed with dried pomegranate concentrate powder (PCP) extracts containing 0.90 mg/g of ellagic acid show increased serum estradiol and bALP levels with decreased serum osteocalcin compared with OVX control rats [85]. The failure load of the femur was also significantly increased, suggesting that the PCP treatment activates osteoblast differentiation and inhibits bone mineralization and turnover. *In vitro*, ellagic acid induces nodule mineralization in an osteoblastic cell line (KS483) through a pathway involving the oestrogen receptor [86,87]. However in our *in vivo* experiment, we did not observe any difference in uterine weight between the OVX control and the OVX PGPE groups. This result could be explained by the lower dose [83].

In addition, looking beyond the traditional bone perspective, because inflammation and oxidative stress play a major role in the modulation of bone remodelling, [47,88], we investigated the expression of major markers of such processes using Taqman Low density Arrays. We showed that PGPE consumption lowers pro-inflammatory markers and enzymes implicated in reactive oxygen species, ROS synthesis expression (Chemokine ligand 2, CCL2; Interleukin 1 receptor 1, IL1-R1; nitric oxide synthase 2, NOS2 and NADPH oxidase 4, NOX4) and enhances the expression of anti-inflammatory markers and the enzymes implicated in the anti-oxidant process (Interleukin 1 receptor antagonist, IL1-Rn; glutathione peroxidase, GPx; glutathione reductase, GSR). The flavonoid content and their derivatives (rutin, gallic acid, ellagic acid and punicalagin) of the peel fraction of pomegranate have been frequently utilized as antioxidants in various dietary supplements [76,89]. *In vivo*, a pomegranate extract diet rich in ellagitannins has been acknowledged to suppress inflammation by decreasing the IL-6 level in the joints of collagen-induced arthritis in mice [90], while the consumption of pomegranate decreases oxidative status in patients with rheumatoid arthritis [91]. *In vitro*, it has been shown that the hydrolysable tannin punicalagin from dried pomegranate peels may have an anti-inflammatory effect on bone metabolism by inhibiting PGE2 production and cyclooxygenase 2, COX-2 expression in lipopolysaccharide, LPS-induced RAW 264.7 cells [92] and/or repressing osteoclast differentiation through the downregulation of Nuclear Factor Of Activated T-Cells, NFATc1 expression and decreased phosphorylation of the Akt and JNK pathways [93]. Likewise, the anti-inflammatory and antioxidant effects of urolithins have been extensively demonstrated *in vitro* [94,95].

In summary, our study demonstrates that PGPE metabolites can directly modulate bone cell differentiation, leading to an improved resorption/formation ratio together with anti-inflammatory and anti-oxidative effects in the bone microenvironment. This ability can explain the improvement in bone mass and microarchitecture in our model of osteoporosis. These encouraging data suggest that pomegranate consumption could be a promising alternative and complementary therapeutic agent for the prevention of osteoporosis. Nevertheless, more studies are still warranted to further determine the molecular mechanisms involved and to investigate whether these *ex vivo* and preclinical data can be extrapolated to the human situation.
